# Compensatory conservation measures for an endangered caribou population under climate change

**DOI:** 10.1038/s41598-018-34822-9

**Published:** 2018-11-06

**Authors:** Sarah Bauduin, Eliot McIntire, Martin-Hugues St-Laurent, Steven G. Cumming

**Affiliations:** 1 Université Laval, Département des sciences du bois et de la forêt, Pavillon Abitibi-Price. 2405 rue de la Terrasse, Québec, QC G1V 0A6 Canada; 2 Centre d’Ecologie Fonctionnelle et Evolutive (UMR 5175), CNRS. 1919 route de Mende, 34293 Montpellier, France; 3 Pacific Forestry Centre, Natural Resources Canada. 506 Burnside Road West, Victoria, BC V8Z 1M5 Canada; 4 Université du Québec à Rimouski, Département de biologie, chimie et géographie, Center for Northern Studies, Center for Forest Research. 300 allée des Ursulines, Rimouski, QC G5L 3A1 Canada

## Abstract

Future human land use and climate change may disrupt movement behaviors of terrestrial animals, thereby altering the ability of individuals to move across a landscape. Some of the expected changes result from processes whose effects will be difficult to alter, such as global climate change. We present a novel framework in which we use models to (1) identify the ecological changes from these difficult-to-alter processes, as well as (2) the potential conservation measures that are best able to compensate for these changes. We illustrated this framework with the case of an endangered caribou population in Québec, Canada. We coupled a spatially explicit individual-based movement model with a range of landscape scenarios to assess the impacts of varying degrees of climate change, and the ability of conservation actions to compensate for such impacts on caribou movement behaviors. We found that (1) climate change impacts reduced movement potential, and that (2) the complete restoration of secondary roads inside protected areas was able to fully offset this reduction, suggesting that road restoration would be an effective compensatory conservation action. By evaluating conservation actions via landscape use simulated by an individual-based model, we were able to identify compensatory conservation options for an endangered species facing climate change.

## Introduction

Both mitigation and compensatory measures have been applied to biological conservation in the face of exogenous processes such as climate or land use change^1,2^. Mitigation measures seek to avoid or minimize the direct, negative impacts of such processes. Compensatory measures target potential changes, with the goal of offsetting their impacts^3^. However, the direct negative impacts of some processes may be too severe to mitigate^3^. Climate change mitigation, for example, is hard because the changes now underway have high inertia with trajectories that are difficult and slow to alter^4^. Compensatory measures would seek to help affected populations deal with climate change consequences such as assisted migration^5^ or environmental restoration^6^. Compensatory measures may be more effective in this case, and more tractable from the perspective of regional authorities pursuing specific conservation goals. In spite of the critical need for conservation via compensatory measures, we do not have a framework for identifying them and quantifying their efficacy. Here, we explore compensatory conservation measures for climate change impacts on a threatened population of a widely ranging large mammal species: the boreal caribou (*Rangifer tarandus caribou*) population in Gaspésie, Québec, Canada.

Incorporating climate change into conservation studies is emerging as a major challenge in designing new protected areas and other conservation measures^7–9^. For example, habitat protection^10^ and restoration^11,12^ are two possible measures that are used frequently to address current conservation problems. They may also compensate for negative changes anticipated under climate change. However, the effectiveness of particular conservation measures in compensating for undesired changes in population characteristics of interest will likely require models of sufficient mechanistic details and sophistication to evaluate non-linear responses outside of historical conditions. Spatially-explicit individual-based models (SE-IBMs) are promising for such purposes. SE-IBMs can link individual behaviors to landscape features that may be affected by climate change, human land use, as well as conservation measures. For example, in the case of habitat alteration caused by roads, many mammal species exhibit complex movement behaviors in response to the presence of these linear features^13,14^.

For large mammals, loss of habitat and anthropogenic linear features are key drivers of many conservation problems. Establishment of protected areas has been shown to be effective in many cases as it prevents further habitat losses or creates a place for habitat recovery^15^. Similarly, studies have shown that linear features such as secondary roads are heavily used by predators^16,17^ and, at high density, may increase predator-prey encounter rates^18^ and consequently prey mortality^19^. Thus, land rehabilitation through road removal (hereafter “road restoration”) could further benefit endangered prey species, like caribou, by reducing predation rates, a known factor in our study population’s decline^20^. Their impacts on populations are numerous^21^, depending on the size of the road: difficulty of crossing due to fences and light^22^, increased probability of predator presence^18^, and risk of collisions. Secondary roads (e.g., forestry roads) have the potential to be restored, and on primary roads (e.g., highways) over or under-passes can be installed (e.g., Banff National Park^23^). We do not know how effective these two major conservation measures are, or to what degree they are able to compensate for predicted loses due to climate change.

In this study, we propose a simple framework illustrated with a case study to assess the effectiveness of compensatory conservation measures. This involves explicit coupling of efforts at quantifying climate change impacts with efforts at identifying and quantifying conservation measures that might have a similar magnitude, but opposite, impacts. By doing this, we explicitly identify conservation measures that can compensate for undesired changes due to future climate. For our case study, we were motivated by the need for species to move to follow their habitat as it changes with climate and land management and to allow or enhance mixing between disjunct populations. We therefore examine different movement behaviors, among which the movement potential, of an endangered population within a landscape. “Movement potential” has been used in two contrasting ways in the recent literature: by Gustine *et al*.^24^ to refer to an estimated area accessible from a location given observed movement rates; and by Hooten *et al*.^25^ as a potential field whose value at a point is the preferred direction of movement, determined by the gradient of environmental conditions. For the purposes of this study, we define “movement potential” as the aggregate of individuals’ capability and propensity to change their spatial location. Capability depends on individuals’ physical ability and the spatial configuration of their environment^26^. Propensity reflects individuals’ desire to leave or reach certain places^26^. Climate change and anthropogenic activities can affect movement potential^27,28^ by altering environmental conditions^20^ thus changing either individual’s capability^29^ or propensity^30^ to move.

We explore the capacity of compensatory conservation to minimize climate change impacts by using a sufficiently mechanistic SE-IBM to predict animal movement potential on landscapes impacted by land use and climate change. We used the endangered Atlantic-Gaspésie caribou, a relict population now confined to high-elevation habitats within a highly modified, managed boreal forest landscape in Québec, Canada. As elsewhere in North America^31,32^, this caribou population is negatively affected by the presence of roads and a long history of forest management^16,20^. We used a scenario approach to represent the effects of climate change and conservation actions on the structure of future landscapes. Conservation actions included establishing new protected areas and the restoration into natural habitats of secondary road segments inside protected areas, two measures that are actionable by local authorities and that might be expected to increase movement potential.

## Methods

### Compensatory conservation framework

We propose a simple framework to address compensatory conservation questions. First, the conservation problem must have a “difficult-to-alter” cause, such as global climate change. Second, we must identify one or more population characteristics that are likely to be affected by climate change. Third, there must be feasible alternative conservation measures that can counteract the effects of climate change on the identified population characteristics. Fourth, we must have models of sufficient mechanistic sophistication to assess population responses to both climate change and the alternative conservation measures tested. Finally, using the models, we evaluate the consequences of varying levels of implementation of the alternative conservation measures to determine their effectiveness and what level, if any, may suffice to compensate for negative impacts of climate change.

Following the framework, we select: (1) an endangered boreal caribou population whose habitat is threatened by climate and (2) distance and time spent outside of home ranges, use of proposed new protected areas and movement potential as population characteristics that interacts with landscapes and would therefore show a response to both climate and habitat restoration. (3) This population has at least two landscape-scale conservation measures that could have positive impacts: creation of new protected areas and road restoration; (4) there is an SE-IBM available with sufficient sophistication to respond to changes in climate and to alternative conservation measures, and (5) there is ample potential in both conservation measures to examine conservation intensity. We elaborate below.

### Case study: the Atlantic-Gaspésie caribou

**Figure 1:**
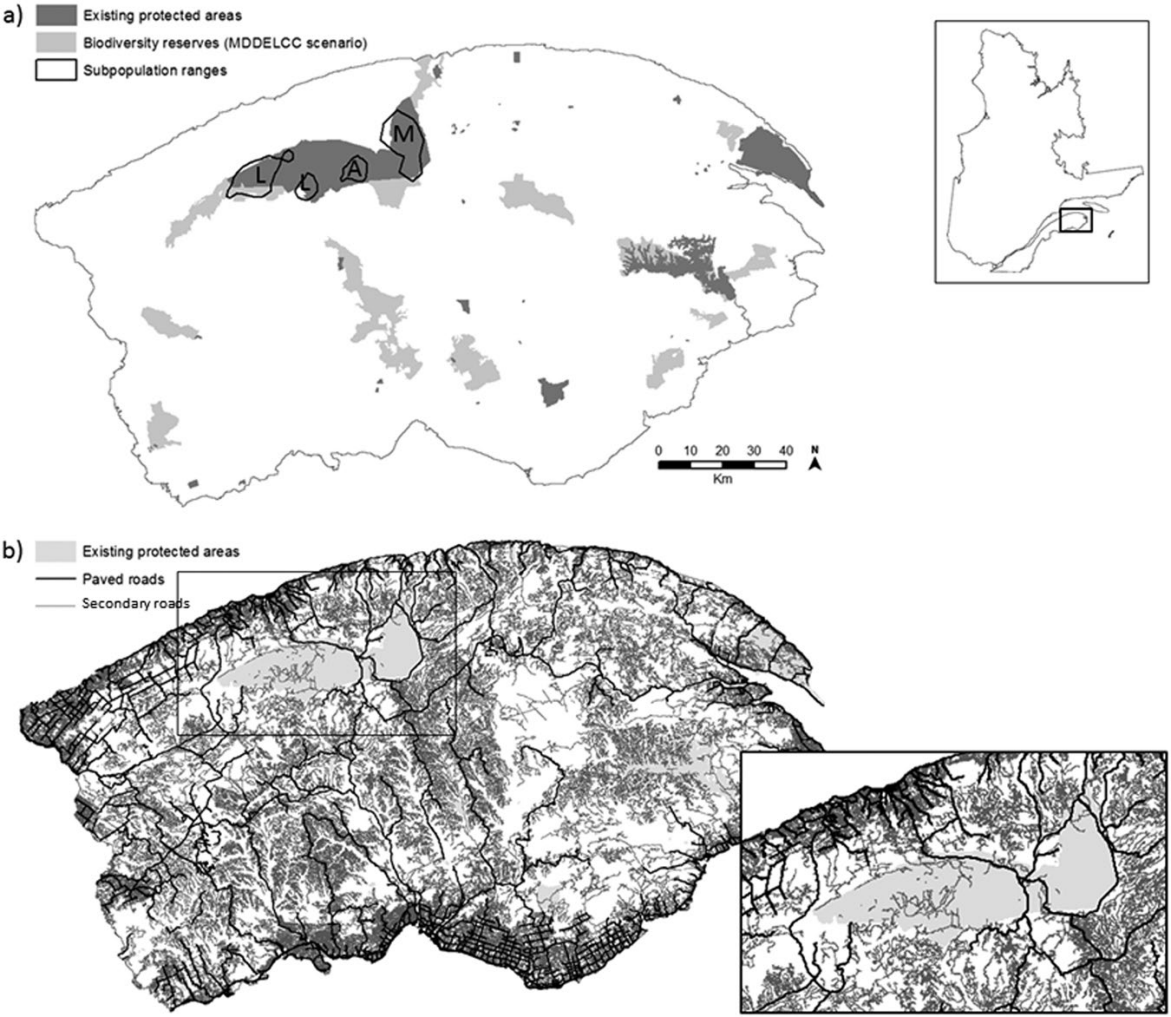
Details of the study area. Upper right inset: location of study area within Québec, Canada. (**a**) Study area (≈25,000 km^2^) on the Gaspésie peninsula, with existing protected areas and proposed new biodiversity reserves, and the ranges of the three caribou subpopulations (L = Logan, A = Albert and M = McGerrigle). The Gaspésie National Park, in the north-west, is the largest protected area (802 km^2^). Caribou ranges were delineated by an 80% kernel density of VHF and GPS locations. (**b**) Road network in the study area, overlaid on the existing protected areas. The road network within and adjacent to Gaspésie National Park is shown in the lower right inset.

Our study system is the caribou population inhabiting the Gaspésie peninsula of Québec, Canada (Fig. 1). The Atlantic-Gaspésie population is the last surviving caribou population south of the St-Lawrence River. Currently, individuals are mostly found within the Gaspésie National Park^33,34^. The population is spatially divided into three subpopulations, associated with breeding grounds on the summits of Mounts Logan, Albert and McGerrigle (Fig. 1a). There have been very few recorded exchanges of individuals among subpopulations^33^, but it is known to occur.

The population has declined in the past several decades and the estimated herd size in 2017 was ≈75 individuals^34^. Consequently, the population has been listed as endangered since 2000^35^ and based on its genetic diversity, movement, behavior and distribution, it was identified as one of the 12 Designatable Units for caribou in Canada (i.e., a significant unit that is an irreplaceable component of Canada’s biodiversity)^36^. Populations of coyotes (*Canis latrans*) and bears (*Ursus americanus*) are sustained by a high abundance of their primary prey species (e.g., moose, *Alces americanus*) and of forage promoted by forestry activities. Incidental predation on caribou by these predators seems to be the main cause of recent declines in the caribou population^20^. The caribou population relies on the alpine tundra as an open space to find mating partners and avoid predators. These caribou primarily feed on alpine herbaceous plants and graminoids. In winter, caribou prefer lower elevation, old stands of balsam fir (*Abies balsamea*) and forage on arboreal lichen^33^. Both these habitats are threatened by climate change^37–39^, likely decreasing habitat quality for the caribou in Gaspésie and further threatening this fragile population.

The Gaspésie peninsula belongs to the boreal biome. Most of its area, except along the coasts, belongs to the balsam fir – white birch bioclimatic domain^40^. The forests are dominated by balsam fir and white spruce (*Picea glauca*) stands mixed with white birch (*Betula papyrifera*) on mesic sites. On less favorable sites, black spruce (*Picea mariana*), jack pine (*Pinus banksiana*) and larch (*Larix spp*) are found alongside white birch or trembling aspen (*Populus tremuloides*). The main natural disturbances in this region are spruce budworm (*Choristoneura fumiferana*) outbreaks^40^ and windthrow (source: ecoforestry maps, Ministère des Forêts, de la Faune et des Parcs du Québec, MFFP). Precipitation is abundant due to the maritime climate and therefore wildfires are infrequent^40^. Outside protected areas, forestry activities are the main disturbances in terms of annual area affected^41^. Most of the forests are publicly owned and managed by MFFP. An extensive network of secondary roads (56,775 km inside the study area so a road density of 2.3 km/km^2^), mostly gravel surfaced, has been constructed to support forest harvesting (Fig. 1b). Some of these secondary roads are present inside protected areas (806 km) with a few in the Gaspésie National Park (376 km), mostly between the Logan and Albert summits (Fig. 1b). A sparser network of primary paved roads (8,116 km) serves the interior of the peninsula with two nearly parallel segments bisecting the Gaspésie National Park and others circling around the park, completely isolating the eastern-most McGerrigle subpopulation from the outside of the park and from the other two subpopulations (Fig. 1b).

Current protected areas where forestry activities are forbidden (IUCN classes I, II and III^42^) cover 1355 km^2^ (≈5.5%) of our study area (Fig. 1a). The “Comité sur les aires protégées terrestres de la Gaspésie” (Ministère du Développement Durable, de l’Environnement et de la Lutte contre les Changements Climatiques du Québec, MDDELCC) is working to increase land protection in this region to 12%. According to their scenario of September 2014^43^, they plan to designate an additional 1,705 km^2^ of biodiversity reserves that would act as new protected areas (Fig. 1a) where no forestry activities would be allowed. Some of these reserves are adjacent to Gaspésie National Park.

### Movement model

In Bauduin *et al*.^44^, we reported how we used low resolution data to estimate parameters for a high resolution SE-IBM simulating the movement of the Atlantic-Gaspésie caribou population, by applying Pattern Oriented Modeling technique^45,46^. The model fitting procedure relied on VHF data collected from 1998 to 2001^33^. Since that time, more intensive high resolution location data for this caribou population became available, from 43 individuals fitted with GPS collars^47^. We used these new data to re-fit the existing SE-IBM^44^ in order to improve the model predictive power. Details of the model and the re-parameterization are given in Supplementary Information S1.We used this revised SE-IBM to simulate and map caribou movement potential on different landscape scenarios, representing future conditions under alternate climate and conservation scenarios. The SE-IBM simulates daily caribou movement using two different habitat-dependent movement behaviors, a random walk in high quality habitats and a foray loop^48^ in low quality habitats. Movement direction is habitat-mediated in both movement behaviors with a preference for higher quality habitats, an avoidance of major paved roads and a seasonal attraction to individuals’ mating area^44^. Habitat quality was defined by two seasonal Resource Selection Functions (RSF) previously developed for the Atlantic-Gaspésie caribou^16^, reflecting the relative probability of occurrence of collared animals (i.e., proxy for the habitat suitability for the population in the study area). RSF models defined habitat conditions using four classes of land cover: alpine tundra, mature fir stands (older than 50 years), regenerating stands (younger than 30 years) and “other”, and three types of linear structures: paved roads, secondary/gravel roads (Fig. 1b) and hiking trails. A predicted RSF value was used as a threshold to separate high and low quality habitats^44^ to then assign the appropriate movement behavior. High quality habitats were mostly alpine tundra on mountain tops or mature fir stands, both usually far from roads and trails. Low quality habitats were mostly regenerating stands and linear features, mainly secondary roads^16^ (Fig. 1).

### Landscape scenarios

We test the efficacy of new protected areas and of restoration of secondary roads inside protected areas as alternative compensatory measures. The movement model has sufficient mechanistic complexity to respond to these two conservation measures which both create more high quality habitats. They will affect the choice of movement behavior and the movement direction of the simulated caribou. In the model, only paved roads act as strong barriers, represented by specific model parameter for road-crossing probability estimated from movement data. These roads were considered too important to the regional economy to be restored into natural habitat and they are not considered in the conservation measures.

We defined 24 landscape scenarios representing possible conditions in 2080 under all combinations of the four climate change scenarios and six conservation scenarios described below. We applied different rules to generate the corresponding future states of the land cover and linear structures layers used in the RSF models. More complete definitions of each scenario and of the rules applied to derive the future landscapes from present conditions are presented in Supplementary Information S2. Scenario building was done using ArcGIS 10.2.2 and R 3.2.0^49^.

### Climate scenarios

Four climate scenarios were defined in terms of impacts with different intensities on vegetation succession and on natural disturbances up to 2080. We used predictions from the literature to generate the future states of each habitat type under each scenario. Scenario CC0 represented the current climate. The magnitude of climate effects varied among the other three scenarios from low (CCMin), to medium (CCMed) to high (CCHigh). Overall, climate change impacts reduced the areas of tundra, lost through shrub densification^39,50^. Climate change negatively impacted mature fir stands by reducing the species potential niche^38^ but also positively by decreasing the severity of spruce budworm outbreaks^51,52^. Consequently, the lesser impact of this natural disturbance resulted in a lower proportional area of regenerating stands.

### Conservation scenarios

We created six conservation scenarios as a combination of two scenarios of protected areas and three scenarios of road restoration.

#### Protected area scenarios

PA0 represented the current state with the existing protected areas in the landscape (Fig. 1a). In scenario PA+, we added the biodiversity reserves defined by the MDDELCC as new protected areas (Fig. 1a). Within protected areas, forest stands were affected by natural disturbances and climate change when predicting future conditions. Outside protected areas, forestry activities and climate change impacted forest stands. We applied the governmental forest management plan (source: Bureau du forestier en chef) to predict the future forest cover. Current management actions try to limit natural disturbance impacts on forests outside protected areas and, furthermore, forest management planning implicitly includes them^53^. Thus, we did not explicitly include natural disturbance impacts outside protected areas.

#### Road restoration scenarios

We tested the potential of land restoration to reduce the negative effects of roads as another potential conservation activity. We represented road restoration scenarios in the model by reducing the density of secondary roads inside protected areas (Fig. 1b). This can change movement behavior by locally improving the habitat quality of presently roaded areas. We defined three road restoration scenarios. No roads were modified in Road0, we reduced road density by 50% in Road50 and by 100% in Road100. Road restoration treatments applied inside all existing protected areas, and also in the biodiversity reserves under the 12 landscape scenarios including PA+.

### Mapping movement

For each of the 24 landscape scenarios, we built the two seasonal habitat quality layers by applying the RSF models to the scenario’s landscape. We used the updated SE-IBM to run 10,000 independent replicate simulations of caribou movement under each scenario. Simulations were of 4 years duration, included 20 individuals from each subpopulation and were initialized by randomly placing individuals within their respective subpopulation ranges. To incorporate model uncertainty, SE-IBM parameters were sampled from the distributions of the updated parameter estimates (Supplementary Information Table S1.1) for each simulation. We summarized caribou movement by counting the number of caribou visits per landscape cell over the last three years of each simulation. First year locations were not included to limit the effect of initial locations. For each scenario, the per-simulation results were summed over the 10,000 replicates to create predicted movement maps, following Bauduin *et al*.^44^. Initial tests established that increasing the number of individuals, the duration, or the number of replicates per scenario had minimal effect on the results. We also generated a predicted movement map under a null model, as for a 25^th^ scenario. The null landscape was defined by a single, homogeneous habitat quality layer. That is, habitat quality did not vary seasonally or by location and there were no roads; all cells had the same quality value of 1. The null movement model was derived from the SE-IBM by disabling mating site fidelity and foray loop behavior. Therefore caribou movements followed a simple random walk, starting from their initial locations. The resulting predicted movement map resembled an observation of the sum of three independent diffusion models^54^ centered at the subpopulation range centroids.

We used the predictive movement maps to quantify the impacts of the climate change and conservation scenarios on the simulated caribou movement behavior. We assessed three potential impacts: the distance of movement beyond the present subpopulation ranges; the proportion of time spent outside these ranges; and the use of the biodiversity reserves. We measured the movement distance as the number of cells outside the present subpopulation ranges that were visited at least once (Dist_Out_). We used the mean number of visits in these cells to measure the amount of time spent outside the ranges (Use_Out_). We measured the use of biodiversity reserves as the mean number of visits over all cells within the biodiversity reserves (Use_RB_). We tested for scenario effects on these indicators using 3 factor ANOVAs with main effects and all 2-way interactions, without replication. The values calculated for the scenario predictive movement maps are estimates of the expected value of the simulated indicators, given the scenario specifications. The climate and conservation (protected areas and roads) scenarios are represented as treatment factors of 4, 2 and 3 levels, respectively. Therefore, the model degrees of freedom are 6 = 3 + 1 + 2 for the main effects plus 11 = 3 + 6 + 2 for the 2-way interactions, leaving 6 = 24 − 1 − 6 − 11 residual degrees of freedom. In a traditional ANOVA with replications, effects are assessed relative to the process variance, and significance levels decrease with the number of replicates, which can be made arbitrarily large. In ANOVA without replication, effects are assessed relative to a residual variance determined by the experimental design, and significance levels depend on the design but not on the number of replicates per se. Rather, increased replications decreases the standard error of the estimated scenario means. For these reasons, we consider ANOVA on the group means to be appropriate for analysis of simulation experiments.

We calculated movement potential maps under a given scenario as the difference between the predicted movement maps of the scenario and the null model, except for cells containing zeros in both maps were undefined. We then rescaled to [−1; 1] by dividing negative values by the minimum value and positive values by the maximum. Positive values indicate cells into which caribou moved more frequently than under the null model (i.e. cells overused relative to the null model). Negative values indicate underused cells where caribou moved less frequently than under the null model. These maps show areas where caribou have a high or low capability/propensity to move, after correcting for bias due to their initial positions within the subpopulation ranges, and for the effects of distance from these ranges. Overused areas were favored by the caribou due to habitat conditions, road absence or site fidelity; movement to those areas was facilitated and sought by the individuals, and movement potential was therefore high. Underused areas were avoided or unreachable by caribou; movement potential was low. We calculated the area (km^2^) of high movement potential (Area_HMP_) in each scenario, defined as the area where movement potential was greater than 0.01. The results were insensitive to the threshold level above 0.01 but were highly unstable between 0 and 0.01. The threshold of 0.01 removed the noise around zero at locations where the SE-IBM and null model movement maps were similar. We tested the effect of climate and conservation scenarios on Area_HMP_ by a 3 factor ANOVA with main effects and 2-way interactions, as above.

## Results

### Distance moved from subpopulation ranges

There was a significant negative effect of climate scenarios on this indicator (F_3,6_ = 230.7, p < 0.001), relative to the reference scenario CC0. The mean reduction in the indicator relative to CC0 was 7.2%. Our interpretation is that caribou tended to remain closer to their subpopulation ranges in climate impacted landscapes. The effect size was smallest under the intermediate climate scenario (CCMed). There was also some evidence of an interaction between climate and protected areas (F_3,6_ = 5.048, p = 0.04), but the effect sizes were small, with no clear interpretation. The full ANOVA tables for this and the following analyses are presented in the Supplementary Information S3, accompanied by figures of effect size for each factor level and interaction.

### Time spent outside subpopulation ranges

There was a significant effect of climate on this indicator (F_3,6_ = 93.4, p < 0.001). The coefficients were positive for all levels with a mean increase of 6.2%, relative CC0. As with Dist_Out_, the effect size was smallest under the intermediate climate scenario. Overall, climate scenarios led to range contraction (Dist_Out_), partially compensated by increased intensity of habitat use (Use_Out_). There was also a significant positive effect of road restoration (F_2,6_ = 182.5, p < 0.001) and a significant interaction between climate change and road restoration (F_6,6_ = 13.6, p = 0.003). Reducing road density increased use intensity of areas outside the subpopulation ranges. The interaction term suggests that this effect is greatest for scenarios Road100 and increases with the level of climate change.

### Use of biodiversity reserves

There was a significant effect of climate change on the mean number of visits per cell inside the biodiversity reserves (Use_RB_) (F_3,6_ = 28.4, p < 0.001). The effects were negative, and markedly largest under scenario CCHigh. Counter-intuitively, there was also a significant negative effect of protected areas (F_1,6_ = 45.0, p < 0.001). There was also a significant positive interaction between the protected areas and the road restoration scenarios (F_2,6_ = 32.1, p < 0.001). The impact of protected areas was positive under road restoration, and the effect size was markedly highest under full road restoration. It appears that full restoration of secondary roads within existing protected areas increases the accessibility of the new protected areas.

### Movement potential

**Figure 2:**
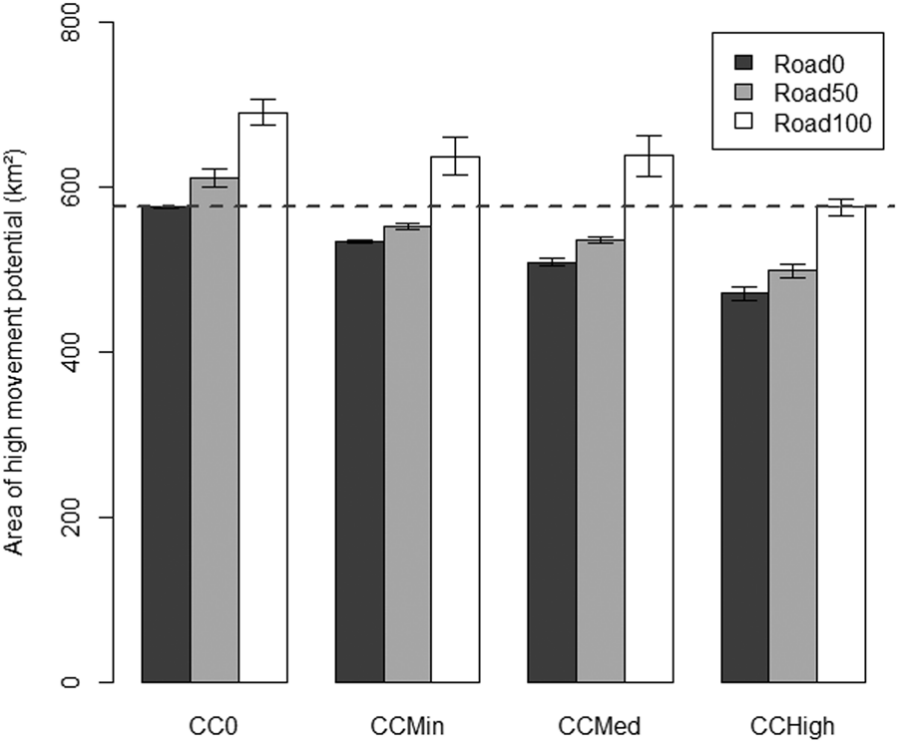
Total area of high movement potential (Area_HMP_) under climate change and road restoration scenarios. The dashed line at Area_HMP_ = 575.8 km² represents the area for the scenario without climate change or road restoration. The values are averaged over the two protected areas scenarios (PA0 and PA+) with the bars representing the 95% confidence intervals on the mean.

There was a significant negative effect of climate change scenarios on the area of high movement potential (Area_HMP_) for caribou (F_3,6_ = 556.5, p < 0.001). As the magnitude of climate change effects increased, Area_HMP_ decreased (Fig. 2). There was a small decreasing effect of the protected area scenarios on Area_HMP_ (F_1,6_ = 12.1, p = 0.013), suggesting that the addition of the new protected areas reduced Area_HMP_. There was a positive impact of secondary road restoration on Area_HMP_ (F_2,6_ = 1245.2, p < 0.001), the more roads were restored, the larger was Area_HMP_ (Fig. 2). Only the scenario with a complete restoration of secondary roads inside protected areas (Road100) was able to fully compensate the impacts of climate change on Area_HMP_ (horizontal dashed line in Fig. 2).There was a significant interaction between the protected areas and road restoration (F_2,6_ = 11.1, p = 0.010).The improvement in movement potential due to the protected areas is enhanced by road restoration as more roads were restored with more protected areas present in the study area.

**Figure 3:**
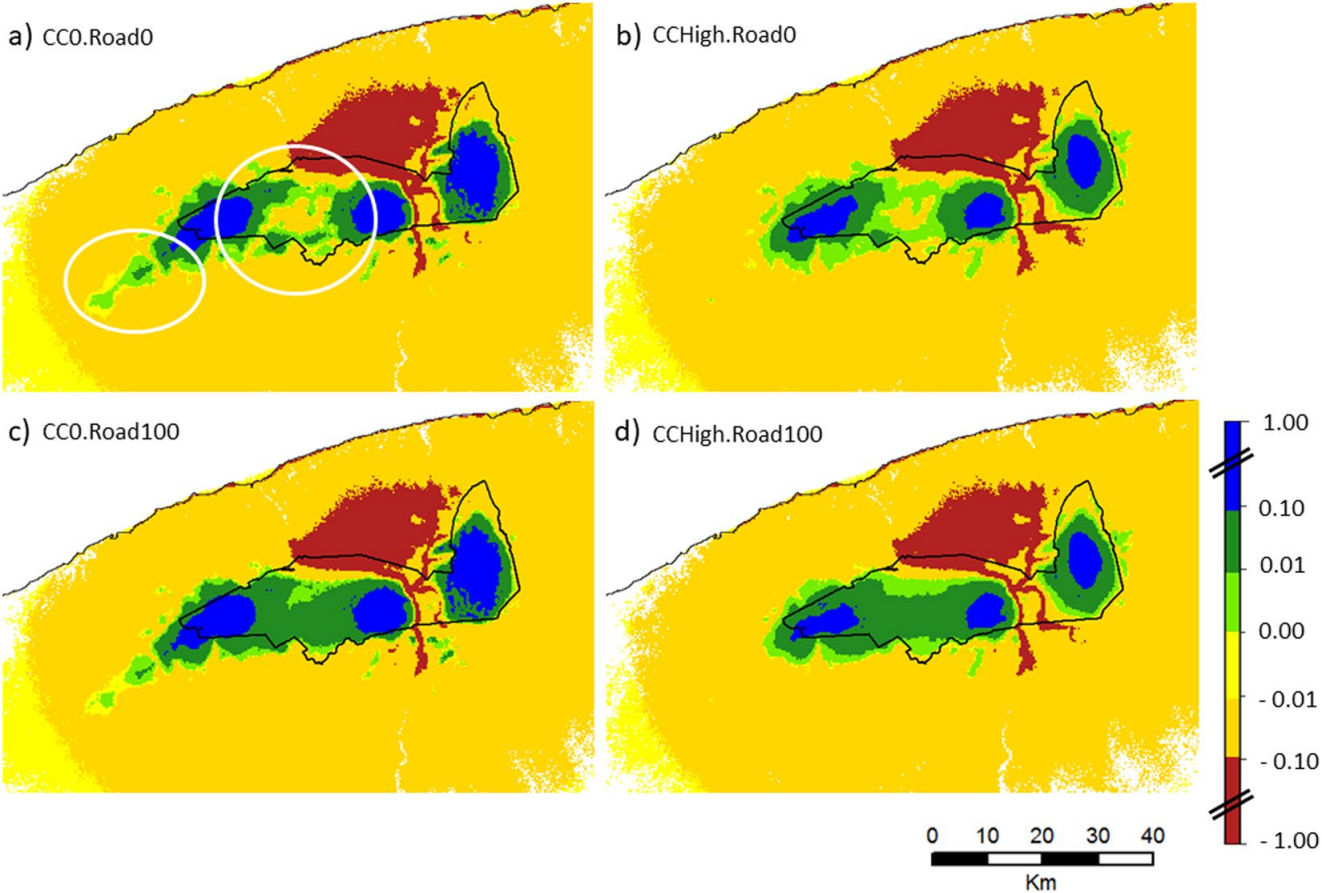
Spatial representation of movement potential for the Atlantic-Gaspésie caribou around the Gaspésie National Park (black outline) for the most extreme climate change and road restoration scenarios. The area shown is similar to the zoomed area presented in the lower right inset of Fig. 1. The color scale represents a gradient of movement potential, from high (blue to green) to low (yellow to red), measured relative to a null model. Values are averaged over the two protected area scenarios (PA0 and PA+). The two white ellipses on (**a**) indicate the regions where differences among scenarios are greatest.

In the base scenario (Fig. 3a), areas with high movement potential were mostly located within subpopulation ranges (Fig. 1a). Some areas outside the Gaspésie National Park were used more frequently than predicted under the null model, mostly areas south and west of the Logan range. Some of these areas were not visited as much under high climate change impacts, and movement was more constrained inside of the park (Fig. 3b). The complete restoration of secondary roads inside protected areas (Fig. 3c) increased the area of high movement potential, mostly by reinforcing travel corridor(s) between the Albert and Logan subpopulations by creating a large patch of high movement potential connecting the two summits. Under the most severe climate change scenario, the complete restoration of secondary roads from Gaspésie National Park (Fig. 3d) was still able to maintain a high movement potential between these two subpopulations inside the park, even though movement potential was still reduced outside of the park. All scenarios identified the same region of very low movement potential associated with the paved roads between the Albert and the McGerrigle ranges (Figs 1 and 3). The McGerrigle subpopulation remained disconnected in all scenarios. Movement potential maps for other scenarios are not shown, as they fell between the four extremes of Fig. 3.

## Discussion

Following the framework we presented, we first identified population characteristics that are likely affected by a high inertia process, here climate change; we then coupled efforts at quantifying climate change impacts on this characteristic with efforts at identifying and quantifying the effects of alternative conservation measures. We chose caribou movement characteristics to inform on the population response to climate change mediated through changes in the landscape. Movement decisions are known to influence individual survival and fitness in caribou^19,55,56^. Other characteristics, such as reproductive success rate, mortality or population structure could be used, as long as they are affected by the identified threat and impact population persistence. The projected effects of climate change on our landscape significantly reduced the use of the landscape far from the caribou ranges and decreased their movement potential. The Atlantic-Gaspésie caribou lives in a mountainous area^33^, where climate change can drive individuals to higher elevation (see examples in Chen *et al*.^57^). Caribou used the reduced area of suitable environment more uniformly. This modification in movement was caused by a change in vegetation cover which reduced the abundance of high quality habitat, outside of the park but also inside, in the heart of their ranges. Climate change is a global threat to biodiversity^58^. Finding effective compensatory measures to help species cope with its impact is an urgent for conservation biology^37^.

The restoration of secondary roads inside protected areas was the most effective of the two compensatory strategies we considered. Roads are an important target for conservation action. Road networks fragmenting landscapes inhibit movements for many species^44,59,60^. Our findings point to the importance of managing secondary road networks for their effects on movement potential, specifically by limiting or reducing the density of such roads. This conservation measure has on-the-ground momentum with a pilot project in Gaspésie recently started to close and rehabilitate derelict roads^61^. Our study is the first to show that this effort, if expanded, could offset predicted declines due to climate change. Restoring secondary roads inside protected areas, and in particular inside the Gaspésie National Park, increased the use of habitat around the subpopulation ranges as a result of a better habitat quality, while keeping the individuals close to their mating areas even in the case of a habitat reduction due to climate change. However, the full restoration of secondary roads was required to fully compensate for movement reductions we predicted under climate change. In this study we simulated road restoration by reducing road density, removing 50% or 100% of the road segments. In practice, what is required is not full road restoration, but rather restoration of functional habitat on roaded areas^62^. The technical requirements and costs for such restoration in our study area could not be considered here. The SE-IBM we used in this study could be applied in a more detailed study to identify priority sections of roads to restore or locate crossing structures^63^ to be most effective and to predict their effect on movement characteristics and subpopulation connectivity. This potential model application would be supported by the clear presence of travel corridors (see Fig. 3a,b).

Implementing the Government of Québec’s proposed new protected areas (Fig. 1a) increased caribou movement potential but was not able to enough compensate for its decline under climate change scenarios. One explanation for this is that most of the proposed new protected areas are located too far from the existing population range to be meaningfully reachable by individual caribou, and so did not increase the area within which they moved. However, these protected areas were visited more frequently when secondary roads were restored. Therefore, a better explanation seems to be that the existing road network inside the proposed protected areas impedes movement sufficiently as to negate their beneficial impact on overall movement potential. Protected areas can be effective tools for species conservation^64^. However, the design of protected areas networks must be adapted to the emerging threat of climate change^9,65^. Our findings show how this could be accomplished for the particular case of caribou in Gaspésie, and support our proposed framework to address the problem in general.

We found that secondary road restoration inside protected areas greatly improved the movement potential between subpopulations. Animal populations constrained in small areas suffer from lack of resources or habitat access^66^, while isolated subpopulations are subject to elevated extinction risk^14^. This increase in movement potential, therefore would likely improve their functional connectivity, increasing the rates of exchange of individuals among subpopulations and thus reducing extinction risk at both the subpopulation and population levels^14,67^. Our results are consistent with several other studies showing that protecting and enhancing functional connectivity can be an effective conservation measures to protect species under climate change^10,28,68^.

Our estimates of the effects of climate change and our vegetation dynamics simulations were relatively simple. This is in part because those are challenging topics, with many potential assumptions with any model that attempts to address them. Thus rather than focusing on the potential impacts of a more sophisticated vegetation dynamics model and climate effect model, we expressed model uncertainty by running multiple scenarios that bracket the range of possible climate and vegetation conditions. This approach is sufficient, provided the scenarios cover a wide range of potential conditions. We used data-driven RSF models to map habitat quality for our IBM. RSF models tend to be less reliable than mechanistic models when extrapolating under novel conditions (see Bauduin *et al*.^44^). This would limit the applicability of our RSF model if the landscape of Gaspésie were to change markedly under climate change, for example if one habitat type were to disappear, or a new one were to emerge. However, no such drastic changes were simulated. Therefore, we feel that having a more mechanistic model to predict habitat quality would not much affect our key findings regarding the effects of compensatory conservation in this particular circumstance, or, more generally, our proposed framework for evaluating such effects.

## Conclusions

We designed a simple framework for evaluating the effectiveness of different conservation actions faced with a diversity of stressors on an ecological system. We present results showing that addressing lower inertia stressors can compensate for the declines caused by higher inertia stressors. To do this, we linked movement behavior to the spatial distribution of habitat elements and estimated the relative effectiveness of alternate compensatory conservation actions in terms of their ability to offset losses of movement due to anthropogenic disturbances and climate change. By linking landscape structure to movement behavior, this approach has the potential to more directly link habitat change to population consequences. This would help managers to identify conservation actions likely to prove effective in sustaining populations in disturbed landscapes and in compensating for further climate and land-use change. Climate change effects may be hard to stop or reverse, but compensatory conservation actions acting on key elements of the landscapes could help species cope with some negative impacts of climate change.

## Electronic supplementary material

Supplementary Information

## Data Availability

The habitat quality layers (raster layers) for each of the 24 scenarios are available at the DRYAD repository, 10.5061/dryad.n726pq6. The source code of the model presented here is available as a SpaDES module (https://github.com/PredictiveEcology/SpaDES-modules/tree/master/modules/caribou2Movements). SpaDES, or Spatial Discrete Event Simulator (https://cran.r-project.org/package=SpaDES), is an ecosystem of R-based spatial simulation models.
