# Supplementary material for: Compensatory conservation measures for an endangered caribou population under climate change
Source: Sci Rep. 2018 Nov 6;8:16438. doi: 10.1038/s41598-018-34822-9 (PMC6219550; doi:10.1038/s41598-018-34822-9)

**Title: Compensatory conservation measures for an endangered caribou population under climate change**

**Authors**: Sarah Bauduin (sarah.bauduin@cefe.cnrs.fr)^a,b,*^, Eliot McIntire (eliot.mcintire@canada.ca)^a,c^, Martin-Hugues St-Laurent (martin-hugues_st-laurent@uqar.ca)^d^, Steven G Cumming (stevec@sbf.ulaval.ca)^a^

^a^ Université Laval, Faculté de foresterie, de géographie et de géomatique, Pavillon Abitibi-Price. 2405 rue de la Terrasse, Québec, QC, G1V 0A6, Canada

^b^ Centre d’Ecologie Fonctionnelle et Evolutive (UMR 5175), CNRS. 1919 route de Mende, 34293 Montpellier, France

^c^ Pacific Forestry Centre, Natural Resources Canada. 506 Burnside Road West, Victoria, BC, V8Z 1M5, Canada

^d^ Université du Québec à Rimouski, Département de biologie, chimie et géographie, Center for Northern Studies, Center for Forest Research. 300 allée des Ursulines, Rimouski, QC, G5L 3A1, Canada.

***Corresponding author**: Sarah Bauduin

Mailing address: Centre d’Ecologie Fonctionnelle et Evolutive (UMR 5175), CNRS. 1919 route de Mende, 34293 Montpellier, France

Phone number: +33.4.67.61.34.34 #33287

Email address: sarah.bauduin@cefe.cnrs.fr

**Supplementary information S1**: Spatially explicit individual-based model (SE-IBM) used to simulate the Atlantic-Gaspésie caribou movement.

We adapted the SE-IBM of Bauduin et al.^1^ to predict and map caribou movement potential under alternate climate and conservation scenarios. The model simulates daily caribou movement over the landscape as a function of behavioral state, season and environmental conditions. Bauduin et al.^1^ previously fit a multiple hypothesis caribou movement model to these landscapes and found best support for caribou that follow either a random walk in high quality habitat or a foray loop^2^ in low quality habitat. The best habitat-mediated movement hypothesis included a preference for high quality habitat, an avoidance of the major paved roads, which are considered as strong movement barriers, and an attraction to individuals’ mating area during mating season to represent site fidelity^1^.

Habitat conditions were defined by a land cover layer and a linear feature layer obtained from digital versions of 1:20,000 ecoforestry maps and associated anthropogenic structures maps produced by Government of Québec (MFFP, http://www.mffp.gouv.qc.ca/forets/inventaire/fiches/carte-ecoforestiere.jsp). The land cover layer distinguished four classes: alpine tundra, mature fir stands (older than 50 years), regenerating stands (younger than 30 years) and “other”. The linear feature layer represented three types of linear structures: paved roads, secondary/gravel roads (main text, Fig. 1b) and hiking trails. The four land cover classes were derived from map attributes of tree species composition, age and disturbance history. Winter and summer habitat quality raster layers (75 x 75 m) were predicted from seasonal RSF models developed for the Atlantic-Gaspésie caribou by Gaudry^3^. These RSF models were defined using a use-availability design conducted at the 2^nd^ order^4^ representing the habitat selection patterns of VHF-collared caribou when facing the land cover types and linear features found available in their habitat. The RSF models are logistical regressions comparing use (VHF recorded positions, coded 1) and available (random points, coded 0) locations. The models included the four land cover classes and the three anthropogenic linear structures described above. We used these models and included the paved roads by creating a raster of distance to the closest paved roads. Then, we extracted the minimum distance for each caribou location and random point. To include the secondary roads and trails, we measured their densities within 1 km circular buffers around each caribou location and random point. The estimated coefficients for the paved road, secondary road and trail covariates in the RSF models indicated an avoidance of all these linear features by the caribou. The RSF models were applied on the landscape to define a RSF score for each cell. These scores were standardized between 0 and 1 and represented relative probabilities of caribou occurrence^3^. We used these standardized scores as landscape quality values. Habitats considered of high quality by the Atlantic-Gaspésie caribou were alpine tundra at the mountain tops and mature fir stands, and far from roads and trails. Low quality habitats were regenerating stands and areas close to roads and trails. Roads affect movement decisions in two ways: roads of all types decrease landscape quality through the RSF model and they also, but only the paved ones, act as strong barriers with associated crossing probabilities that simulated individuals avoid^5,6^.

The SE-IBM of Bauduin et al.^1^ was initially parameterized by pattern-oriented modeling^7,8^ using patterns defined from Very High Frequency (VHF) collar telemetry data. Data represented 35 collared caribou which were located, on average, every two weeks between 1998 and 2001^9^. From these data, three patterns were defined: the emergence of subpopulations ranges, the frequency of paved road crossings and the distributions of distances moved. We used the best-supported model structure identified by Bauduin et al.^1^ but we took advantage of newly available GPS location data of the Atlantic-Gaspésie population to re-fit the model in order to improve its predictive power. The GPS locations came from 22 individuals followed from February 2013 and a further 21 followed from February/March 2014, until November 2014^10^. GPS data was collected in strict accordance with the recommendations of the Canadian Council on Animal Care and both captures and manipulations of study animals were approved by the Animal Welfare Committee of the Université du Québec à Rimouski (certificate #52-13-112). Captures were conducted on public lands, under the supervision of the Québec government (MFFP), so no land use permissions were required. GPS data were subsampled to match the daily resolution of the SE-IBM. We randomly selected one GPS location per day per individual recorded between 2 pm and 6 pm, the hours when most of the VHF data had been obtained. From these sampled GPS data, we defined the same three patterns as Bauduin et al.^1^ did using the VHF data.

We used the best-fit calibration method^11^ as adapted by Bauduin et al.^1^ for parameter estimation of the movement model; we re-fit the model by adding the three patterns defined with the new GPS locations to the original VHF patterns. We treated the VHF and GPS collar data as two independent data sets. We generated 100,000 independent random samples of each parameter to be estimated by sampling from uniform distributions on parameter-specific intervals, as defined in Bauduin et al.^1^. For each sample, we simulated movements of the collared individuals for lengths of time consistent with the respective survey periods (i.e., VHF and GPS data survey periods) and extracted the patterns according to these periods for comparison with the ones defined from the field data. The seasonal habitat quality layers of the model were predicted using the RSFs and the land cover data that were temporally closest to the times when the collar data were collected. When recreating movements of the VHF-equipped caribou, we used land cover data from the ecoforestry maps of the 3rd forest inventory program (updated in 1998). We used maps from the 4^th^ inventory program (updated in 2009) when simulating movements of the GPS-equipped caribou. We used the same pattern-oriented modeling strategy as in Bauduin et al.^1^ to compare simulation outputs to the telemetry data. We selected the simulations which produced movement patterns most similar to those defined from the VHF and the GPS data, up to selecting 0.5% of all simulations (representing 470 simulations). Parameter values were estimated using these simulations following Bauduin et al.^1^. We used kernel density estimators to determine the parameter estimates and an Efron bootstrapping method to obtain their 95% confidence intervals (Table S1.1). These parameter values were used to simulate caribou movement for this study. Parameter estimation and all model runs were conducted in R 3.2.0^12^. Simulation experiments were parallelized using an HTCondor cluster^13^.

**Table S1.1**: Parameter estimates and their 95% confidence interval for the updated caribou spatially explicit individual-based model.

| Parameter | Estimate | 95% confidence interval | Unit |
| --- | --- | --- | --- |
| Mean step length in in good quality habitat *µ_sl.good_* | 5.47 | [5.21;5.65] | log(m) |
| Mean step length in low quality habitat *µ_sl.low_* | 5.74 | [5.44;6.08] | log(m) |
| Standard deviation of the step length *σ_s_* | 1.20 | [0.90;1.43] | log(m) |
| Probability of crossing a paved road *p_cross_* | 0.10 | [0.09;0.12] |  |
| Standard deviation of the truncated Normal distribution for the mating area attraction *σ_ma_* | 42.62 | [28.18;68.69] | degrees |
| Standard deviation of the truncated Normal distribution for the foray loop movement *σ_fl_* | 83.48 | [77.85;131.74] | degrees |
| Maximum step length of the outgoing portion of the foray loop *max_steps.loop_* | 3.16 | [2.45;8.44] | steps |

**References**

1. Bauduin, S., McIntire, E. J. B., St-Laurent, M.-H. & Cumming, S. G. Overcoming challenges of sparse telemetry data to estimate caribou movement. *Ecol. Modell.* **335,** 24–34 (2016).

2. Conradt, L., Zollner, P. A., Roper, T. J., Frank, K. & Thomas, C. D. Foray search: an effective systematic dispersal strategy in fragmented landscapes. *Am. Nat.* **161,** 905–15 (2003).

3. Gaudry, W. Impact des structures anthropiques linéaires sur la sélection d’habitat du caribou, de l’ours noir et du coyote en Gaspésie. (Université du Québec à Rimouski, 2013).

4. Johnson, D. H. The comparison of usage and availability measurements for evaluating resource preference. *Ecology* **61,** 65–71 (1980).

5. Polfus, J.L., Hebblewhite, M. & Heinemeyer, K. Identifying indirect habitat loss and avoidance of human infrastructure by northern mountain woodland caribou. *Biol. Cons.* **144**, 2637–2646 (2011).

6. Beauchesne, D., Jaeger, J.A.G. & St-Laurent, M.-H. Disentangling woodland caribou movements in response to clearcuts and roads across temporal scales. *PLoS ONE* **8**, e77514 (2013).

7. Grimm, V. *et al.* Pattern-oriented modeling of agent-based complex systems: lessons from ecology. *Science (80-. ).* **310,** 987–991 (2005).

8. Grimm, V. & Railsback, S. F. Pattern-oriented modelling: a ‘multi-scope’ for predictive systems ecology. *Philos. Trans. R. Soc. London - Ser. B* **367,** 298–310 (2012).

9. Mosnier, A., Ouellet, J., Sirois, L. & Fournier, N. Habitat selection and home-range dynamics of the Gaspé caribou: a hierarchical analysis. *Can. J. Zool.* **81,** 1174–1184 (2003).

10. Lesmerises, F., Johnson, C. J. & St-Laurent, M.-H. Refuge or predation risk? Alternate ways to perceive hiker disturbance based on maternal state of female caribou. *Ecol. Evol.* **7,** 845–854 (2017).

11. Railsback, S. F. & Grimm, V. *Agent-based and individual-based modeling: a practical introduction*. (Princeton University Press, 2012).

12. R Core Team. R: A language and environment for statistical computing. (2014).

13. Thain, D., Tannenbaum, T. & Livny, M. Distributed computing in practice: The Condor experience. *Concurr. Comput. Pract. Exp.* **17,** 323–356 (2005).

**Supplementary information S2:** Construction of potential future landscapes according to the climate change and landscape conservation scenarios.

*Climate change scenarios*

We evaluated four different climate change scenarios. CC0 was a scenario without climate change while scenarios CCMin, CCMed and CCHigh represented a gradient of minimum, medium and high climate change impacts. These scenarios represented possible climate change impacts. They did not correspond to any particular climate forecasts such as those of the Intergovernmental Panel on Climate Change (IPCC) assessment reports (e.g., ^1^) as it was beyond the scope of this study to do detailed simulations of the vegetation under the different IPCC scenarios. Climate impact scenarios were defined in terms of their effects on vegetation. The effects were modelled by changing the rules for vegetation succession and disturbances based on the literature. We applied these rules to the current landscape to forecast the potential state in 2080 of alpine tundra, mature fir stands (older than 50 years) and regenerating stands (younger than 30 years) for each scenario. The year 2080 was chosen because of the data availability for vegetation and disturbances, which are rarely forecast further than the time period 2070-2100^2–5^.

*Alpine tundra*

Alpine tundra in Gaspésie is climate driven^6^ with wind being a major factor^7^. Even though no change was observed in the treeline position between 1975 and 2008, researchers noticed a shrub densification of (*Betula glandulosa*) above the treeline and a development of a more erected tree from for some krummholz (*Picea glauca*)^6^. *B. glandulosa* radial growth is positively associated with summer temperatures^6^ and climate models predicted an increase of these temperatures for the Gaspésie peninsula for the horizon 2090^4^. It is therefore possible that alpine tundra may be colonized by upright vegetation (i.e., erect trees) on its rim due to climate change. The tundra was assumed constant under CC0. We shrunk the tundra polygons (as defined in Gaudry^8^) in the scenarios including climate change. No estimation of alpine tundra reduction was available from the literature or experts, so shrinkage amounts were chosen for simplicity and to show significant difference with the current state. For most tundra sites, we buffered by 100, 200 and 500 m in CCMin, CCMed and CCHigh, respectively. The exception was the Mount Albert summit, the plateau in the center of the Gaspésie National Park^9^, which gave the name to subpopulation located there (main text, Fig. 1a). Mount Albert is composed of serpentine^10^ which is less subject to vegetation colonization relative to the other tundra areas. We buffered this area by 50 m, 100 m and 250 m for CCMin, CCMed and CCHigh, respectively.

*Mature fir stands and regenerating stands*

The abundance and distribution of mature fir stands and regenerating stands are driven by both climate and disturbances.

i) Impact of climate change

Due to climate change, the potential habitat of balsam fir is likely to decrease across Québec^5^. Predictions of these changes to 2080 have been mapped over eastern North America on a grid cell of 20 x 20 km (source: Ministère des Forêts, de la Faune et des Parcs du Québec, MFFP). Potential habitat for balsam fir is predicted to be either lost, lower in quality compared to the present or else mostly unchanged. Potential habitat is predicted to increase in quality in some locations, but not in our study area. Using a GIS, we determined the predicted habitat change at the centroid of each fir stand polygon in our study area. We applied a mortality probability which changed the fir stand into “other” when balsam fir potential habitat was predicted to be either lost or to decline in quality. No mortality probability values were available from the literature or experts so we chose values which provided a reasonable range of consequences among our scenarios. No mortality was applied on fir stands for CC0. Mortality probabilities for fir stands where potential habitat conditions were predicted to be lost in 2080 were set at 0.01, 0.10 and 0.50 in CCMin, CCMed and CCHigh respectively. Mortality probabilities in stands where habitat quality decreases were predicted were set at 0.01 and 0.10 in CCMed and CCHigh.

ii) Impact of disturbances inside protected areas

We used the current protected areas and the biodiversity reserves planned by the Quebec government (source: Ministère du Développement Durable, de l'Environnement et de la Lutte contre les Changements Climatiques du Québec, MDDELCC) to represent protected areas in our scenarios. Forest harvesting is excluded from these areas so only rules about natural disturbances were applied to forest stands inside these areas. Forest stands already accounted for climate change impact on fir stands.

The dominant natural disturbances in Gaspésie are spruce budworm outbreaks^11^ and, to a lesser extent, windthrow. Spruce budworm outbreaks have a mean interval frequency of about 40 years in eastern Québec and this frequency has not changed much since the mid-16^th^ century^12^. Our scenarios were evaluated at 2080 so we defined 2000 as the reference year for spruce budworm impacts in the outbreak cycle, corresponding to two outbreak intervals. Analysis of the maps from the 3^rd^ and 4^th^ forest inventories (source: MFFP), spanning more than two decades, indicated that the impact of windthrow, fire and other disturbances were small compared to that of spruce budworm in our study area. We assumed the magnitude and effects of natural disturbances other than spruce budworm outbreaks constant over the simulation interval. Therefore we used 2000 as the reference year for all natural disturbances.

For scenario CC0, we assumed that the forest is in a dynamic equilibrium and so the forest age composition in 2000 inside the protected areas resulting from natural disturbances was used to represent that of 2080. Using the ecoforestry maps, we selected all forest polygons inside the protected areas which were undisturbed by human activities prior to 2000. The age of these stands in 2000 was kept to represent their age in 2080. The few stands inside protected areas affected by human disturbances before 2000 were classed as mature in 2080.

Under climate change, spruce budworm outbreaks in Gaspésie are predicted to be 10-14 years longer and 26-75% less severe over 2080-2100 than at present^2^. Spruce budworm population growth rates are predicted to decline during 2041-2070, under expected condition of climate and forest cover^3^. Accordingly, we assumed that spruce budworm impacts on forest stands would be less than currently under our climate change scenarios. We simulated this by decreasing the proportion of regenerating stands and therefore increasing the proportion of mature stands as a consequence, in the projected landscapes to reflect a reduction of the mortality due to spruce budworm outbreaks. From the landscape created for CC0, we selected the regenerating stands inside protected areas for which spruce budworm outbreak was the recorded disturbance. We randomly sampled some of these forest stands and turned them as mature in 2080. Based on Gray^2^, the proportions of stands sampled were 0.25, 0.50 and 0.75 for scenarios CCMin, CCMed and CCHigh, respectively.

iii) Impact of disturbances outside protected areas

Outside protected areas, the majority of the landscape is managed for timber production (source: Bureau du Forestier en Chef, BFEC). Efforts are made to prevent or combat spruce budworm outbreaks^13^. Damaged wood is salvaged and plantations may be established in affected areas. Losses to budworm defoliation are accounted for in periodic calculations of annual allowable cut (AAC)^13^. Windthrow is similarly managed for^13^. Therefore, outside protected areas, modifications on forest stands could be regarded as mainly due to forest management. We used the forecasts made by the BFEC to represent the forest composition outside protected areas in 2080 and we did not simulate any extra natural disturbances on these stands.

In Québec, public forest lands are spatially stratified into management units. The BFEC develops management plans and calculates AAC for each unit. There are five management units in Gaspésie, covering 72% of the forest outside protected areas. We assigned the small areas of private forests to these management units, based on the stand proximity with each unit. We applied the BFEC plans to these slightly modified units.

Due to ecosystem management practices in Québec, BFEC plans are expected to increase the amount of old forest and slightly decrease the proportion of regenerating forest relative to the present day. Plans also entail a decreased proportion of fir stands in our study area (source: BFEC). We used the per-unit harvest rates and AACs under all scenarios; climate change impact on fir stands was accounted for beforehand. In each unit, we calculated the proportional decreases in the areas of fir and regenerating stands and increases in old forests from 2008 to 2083. These years were the closest matched to the dates of the ecoforestry maps (2005) and the simulation endpoint (2080). Within each unit, we randomly selected fir and regenerating stands up to the indicated proportional area and reclassified them as follows. Fir stands were reclassified as type “other” for their forest type. Regenerating stands were reclassified as “other” for their age category. The BFEC defined regenerating forest stands as those less than 10 years old^13^. We assumed the indicated proportional reductions applied also to our broader definition of regenerating stands, as those younger than 30 years^8^. The BFEC defined old forest as those older than 80 years^13^, whereas we needed to forecast the abundance of mature forest older than 50 years^8^. In each unit, the projected increase in the abundance of old forest exceeded the remaining area of age between 30 and 50 years or undetermined. Accordingly, in simulated landscapes of 2080, all forest stands not explicitly classed as regenerating were classed at mature. This approximation will not affect scenario outputs as the proportion of forests between 30 and 50 years old are expected to be small compared to the other age categories, especially given ecosystem management practices intended to increase the amount of mature forests.

*Landscape conservation scenarios*

We defined two protected areas scenarios (PA0 and PA+) and three road restoration (Road0, Road50 and Road100) scenarios in a factorial design, leading to six different landscape conservation scenarios.

*Protected areas*

Protected areas in PA0 represented the current existing protected areas where no forestry activities were allowed inside (main text, Fig. 1a) and the above rules on vegetation were applied according to these areas. In the scenario PA+, we increased land protection by adding the reserves of biodiversity defined by the MDDELCC (main text, Fig. 1a). The impacts of natural disturbances and forest management as previously defined were applied according to the protected areas defined in the scenario.

*Road restoration*

There are not established plans for future road development in our study area so we kept the current road network to represent the one in 2080. Our simulated landscape restoration scenarios removed secondary road segments. The habitat quality layer used in the caribou movement model recognized three road types^8,14^. Of these, land rehabilitation is most likely to happen on the secondary/gravel road type (main text, Fig. 1b). The network of secondary roads in our study area represents 56,775 km, with 806 km inside the current protected areas, and 2011 km inside the reserves of biodiversity defined by the MDDELCC. In scenario Road0, we did not modify any roads. In Road50, we reduced the density of secondary roads by half inside the protected areas. It represented a removal of 403 km of secondary roads for the land protection scenario PA0, and of 1,409 km for the scenario PA+. In Road100, we completely restored all secondary roads inside the protected areas (i.e., removal of 806 km of secondary roads for the scenario PA0 and of 2,817 km for the scenario PA+). Secondary roads were defined in the RSF models as the density of roads within 1 km buffers so we did not have to choose which road segments to remove in Road50 scenarios. We simply reduced the density values by half. Existing paved roads and trails were not modified in any scenario.

Once the landscapes built with the different rules of climate change and landscape conservation scenarios, we estimated the evolution of the three habitats types we were interested in (i.e., alpine tundra, mature fir stands and regenerating stands). The reference point for the area of each habitat type was the current state as estimated using the 4^th^ forest inventories (source: MFFP). The decrease or increase of the habitat areas were not forced values but the results of the different rules previously described applied to the landscape. For the four climate change scenarios (CC0, CCMin, CCMed and CCHigh), the alpine tundra area remained at 100%, 59%, 39% and 14% respectively. There were no differences among the landscape conservation scenarios as none of the actions tested impacted the alpine tundra. The decrease of the tundra was only driven by climate change. The area of mature fir stands remained at 107%, 107%, 105%, and 91% respectively for the four climate change scenarios and the current protected areas PA0; it remained at 110%, 110%, 109%, and 95% for the scenario PA+. Old stands area increased due to the ecosystem management for the forestry activities, and fir stands area increased due to the reduction of the severity of spruce budworm outbreaks. However, fir stands area decrease as a consequence of the AAC planned over time and climate change which decreased the habitat conditions. The area of regenerating stands remained at 68%, 68%, 68%, and 67% for the four climate change scenarios and the current protected areas PA0; it remained at 67%, 66%, 66%, and 65% for the scenario PA+. Regenerating stands area decreased due to the ecosystem management for the forestry activities and the reduction of the severity of spruce budworm outbreaks.

**References**

1. IPCC. *Fourth Assessment Report (AR4) of the United Nations Intergovernmental Panel on Climate Change*. (2007).

2. Gray, D. R. The relationship between climate and outbreak characteristics of the spruce budworm in eastern Canada. *Clim. Change* **87,** 361–383 (2008).

3. Régnière, J., St-Amant, R. & Duval, P. Predicting insect distributions under climate change from physiological responses: spruce budworm as an example. *Biol. Invasions* (2010). doi:10.1007/s10530-010-9918-1

4. Logan, T. *Scénarios climatiques pour les régions naturelles de la péninsule de la Gaspésie et la dépression de La Tuque*. (2012).

5. Périé, C., de Blois, S., Lambert, M.-C. & Casajus, N. *Effets anticipés des changements climatiques sur l’habitat des espèces arborescentes au Québec*. (2014).

6. Dumais, C., Ropars, P., Denis, M.-P., Dufour-Tremblay, G. & Boudreau, S. Are low altitude alpine tundra ecosystems under threat? A case study from the Parc National de la Gaspésie, Québec. *Environ. Res. Lett.* **9,** 94001 (2014).

7. Renard, S., Isabel, C. & McIntire, E. J. B. Le vent, un facteur déterminant pour le maintien de la toundra alpine. *Bulletin de conservation* 27–30 (2015).

8. Gaudry, W. Impact des structures anthropiques linéaires sur la sélection d’habitat du caribou, de l’ours noir et du coyote en Gaspésie. (Université du Québec à Rimouski, 2013).

9. Mosnier, A., Ouellet, J., Sirois, L. & Fournier, N. Habitat selection and home-range dynamics of the Gaspé caribou: a hierarchical analysis. *Can. J. Zool.* **81,** 1174–1184 (2003).

10. Sirois, L. & Grandtner, M. M. in *The ecology of areas with serpentinized rocks* 115–133 (Springer Netherlands, 1992).

11. Saucier, J.-P., Grondin, P., Robitaille, A. & Bergeron, J.-F. Zones de végétation et les domaines bioclimatiques du Québec. (2003).

12. Boulanger, Y. & Arseneault, D. Spruce budworm outbreaks in eastern Quebec over the last 450 years. *Can. J. For. Res.* **34,** 1035–1043 (2004).

13. Bureau du forestier en chef. *Manuel de détermination des possibilités forestières 2013-2018*. (2013).

14. Bauduin, S., McIntire, E. J. B., St-Laurent, M.-H. & Cumming, S. G. Overcoming challenges of sparse telemetry data to estimate caribou movement. *Ecol. Modell.* **335,** 24–34 (2016).

**Supplementary information S3:** Complete results of the ANOVA analyses with figures showing the effect sizes for all levels of all factors and interaction terms. 3 factor ANOVA with main effects and 2-way interactions to test for effect of scenario (CC = climate change scenarios, PA = protected area scenarios and Road = road restoration scenarios) on indicators of impacts on caribou behavior and landscape movement potential. Reference scenarios for the different effects are CC0, PA0 and Road0. Details of the scenarios CC0, CCMin, CCMed, CCHigh, PA0, PA+, Road0, Road50 and Road100 are presented in the main text and in Supplementary information S2.

*Distance moved from subpopulation ranges*

Dist_Out_ = Distance away from the ranges calculated as the number of cells that were visited at least once outside of the subpopulation ranges.

Dist_Out_ ~ CC + PA + Road + CC*PA + PA*Road + CC*Road

Df Sum Sq Mean Sq F value Pr(>F)

cc 3 7.612e+10 2.537e+10 230.715 1.38e-06 ***

pa 1 6.723e+07 6.723e+07 0.611 0.4640

road 2 5.233e+08 2.616e+08 2.379 0.1735

cc:pa 3 1.666e+09 5.552e+08 5.048 0.0443 *

pa:road 2 8.960e+07 4.480e+07 0.407 0.6825

cc:road 6 1.922e+09 3.204e+08 2.913 0.1095

Residuals 6 6.599e+08 1.100e+08

---

Signif. codes: 0 ‘***’ 0.001 ‘**’ 0.01 ‘*’ 0.05 ‘.’ 0.1 ‘ ’ 1

*
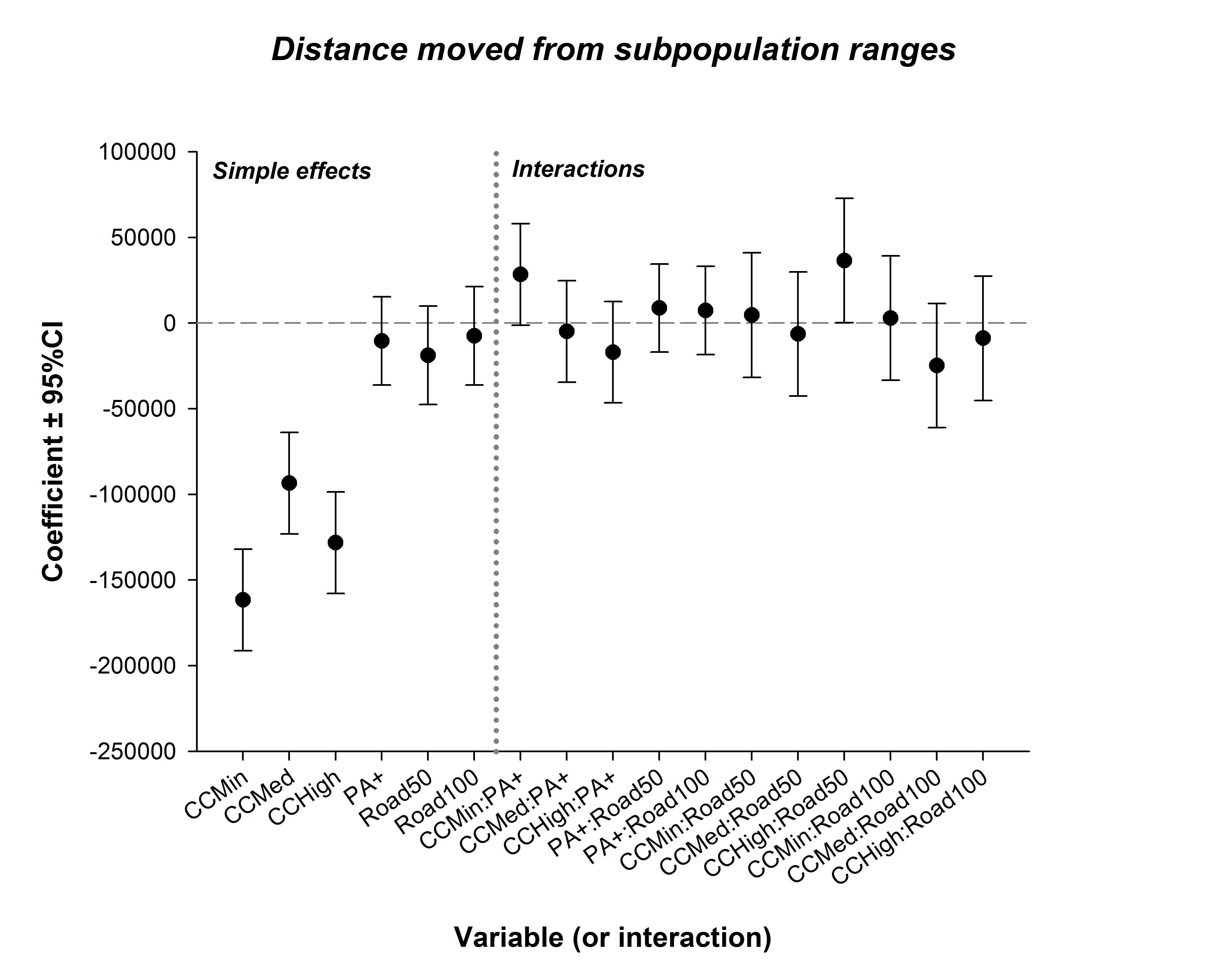
*

*Time spent outside subpopulation ranges*

Use_Out_ = Time spent outside of the ranges calculated the mean number of visits over all cells (excluding the non-visited cells) outside of the subpopulation ranges.

Use_Out_ ~ CC + PA + Road + CC*PA + PA*Road + CC*Road

Df Sum Sq Mean Sq F value Pr(>F)

cc 3 38249 12750 93.381 2.00e-05 ***

pa 1 436 436 3.193 0.12419

road 2 49833 24916 182.494 4.23e-06 ***

cc:pa 3 875 292 2.137 0.19681

pa:road 2 1279 639 4.683 0.05954 .

cc:road 6 11120 1853 13.574 0.00291 **

Residuals 6 819 137

---

Signif. codes: 0 ‘***’ 0.001 ‘**’ 0.01 ‘*’ 0.05 ‘.’ 0.1 ‘ ’ 1

*
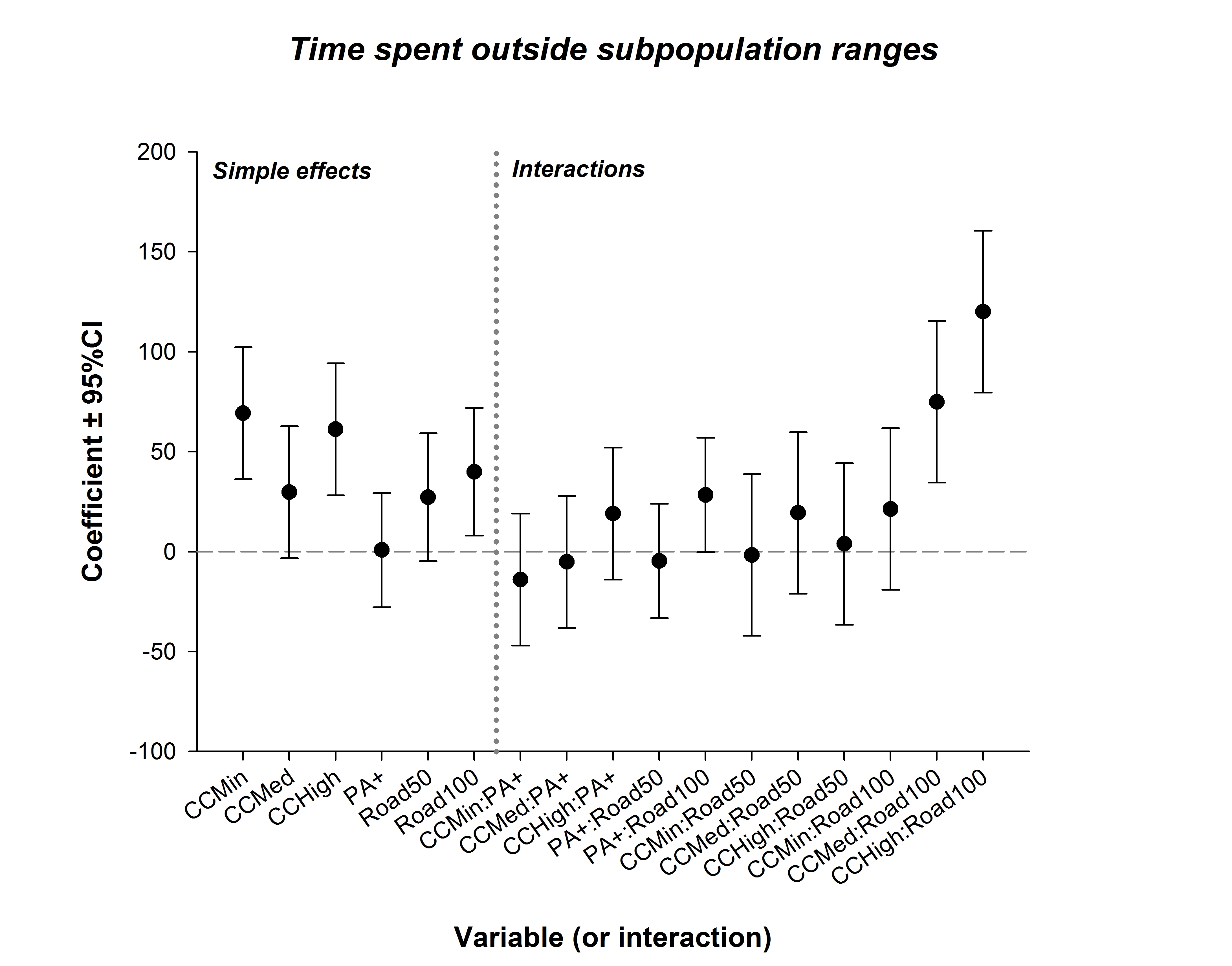
*

*Use of biodiversity reserves*

Use_RB_ = Time spent inside the proposed protected areas calculated as the mean number of visits over all cells inside the biodiversity reserves.

Use_RB_ ~ CC + PA + Road + CC*PA + PA*Road + CC*Road

Df Sum Sq Mean Sq F value Pr(>F)

cc 3 106310 35437 28.430 0.000606 ***

pa 1 56129 56129 45.031 0.000532 ***

road 2 6437 3218 2.582 0.155241

cc:pa 3 2562 854 0.685 0.593028

pa:road 2 80060 40030 32.115 0.000624 ***

cc:road 6 274 46 0.037 0.999580

Residuals 6 7479 1246

---

Signif. codes: 0 ‘***’ 0.001 ‘**’ 0.01 ‘*’ 0.05 ‘.’ 0.1 ‘ ’ 1


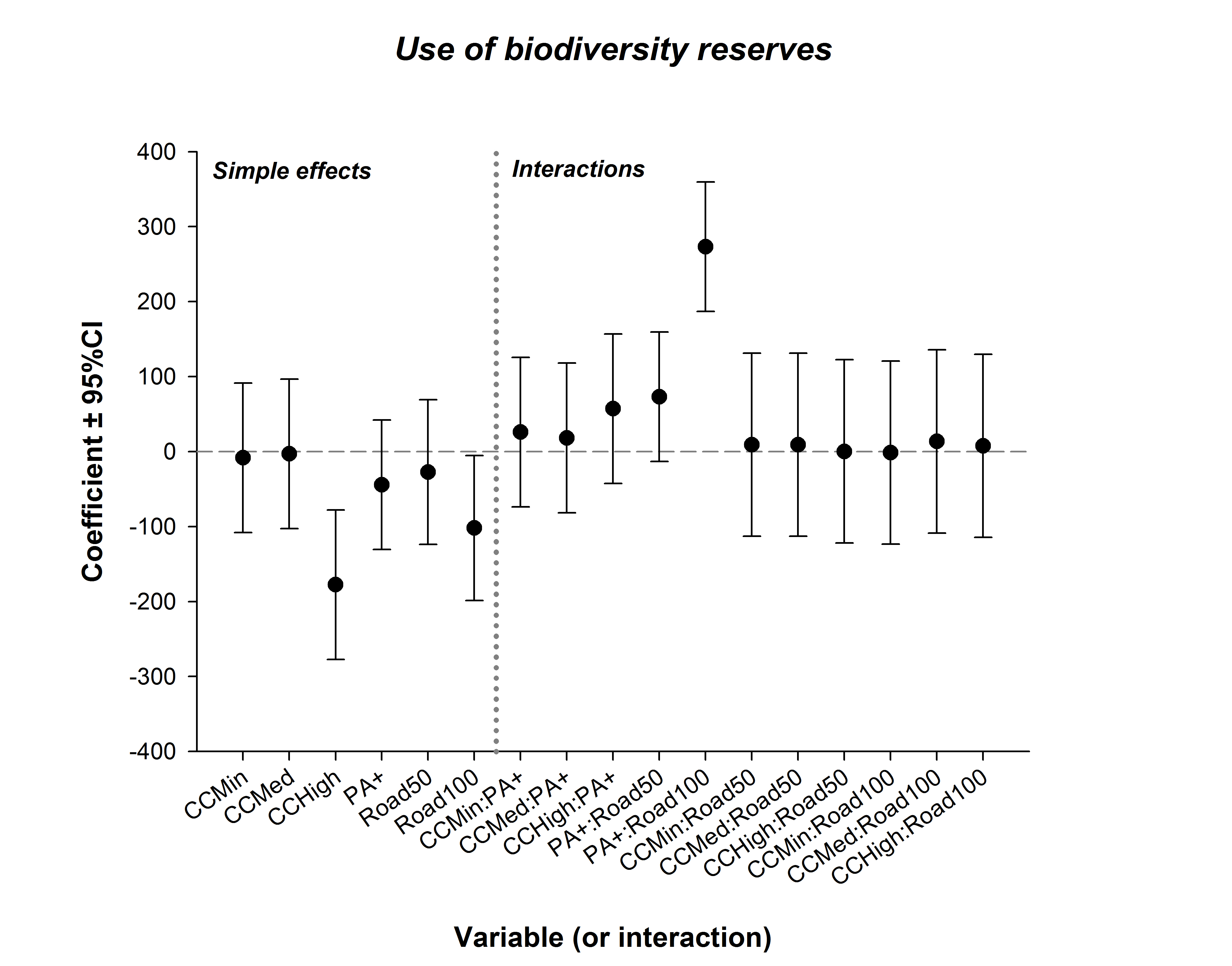


*Movement potential*

Area_HMP_ = Propensity and capability to move across the landscape calculated as the area of relative movement potential greater than 0.01.

Area_HMP_ ~ CC + PA + Road + CC*PA + PA*Road + CC*Road

Df Sum Sq Mean Sq F value Pr(>F)

cc 3 37422 12474 556.458 1.00e-07 ***

pa 1 271 271 12.104 0.01316 *

road 2 55826 27913 1245.192 1.39e-08 ***

cc:pa 3 37 12 0.545 0.66960

pa:road 2 497 249 11.087 0.00966 **

cc:road 6 642 107 4.772 0.03943 *

Residuals 6 135 22

---

Signif. codes: 0 ‘***’ 0.001 ‘**’ 0.01 ‘*’ 0.05 ‘.’ 0.1 ‘ ’ 1


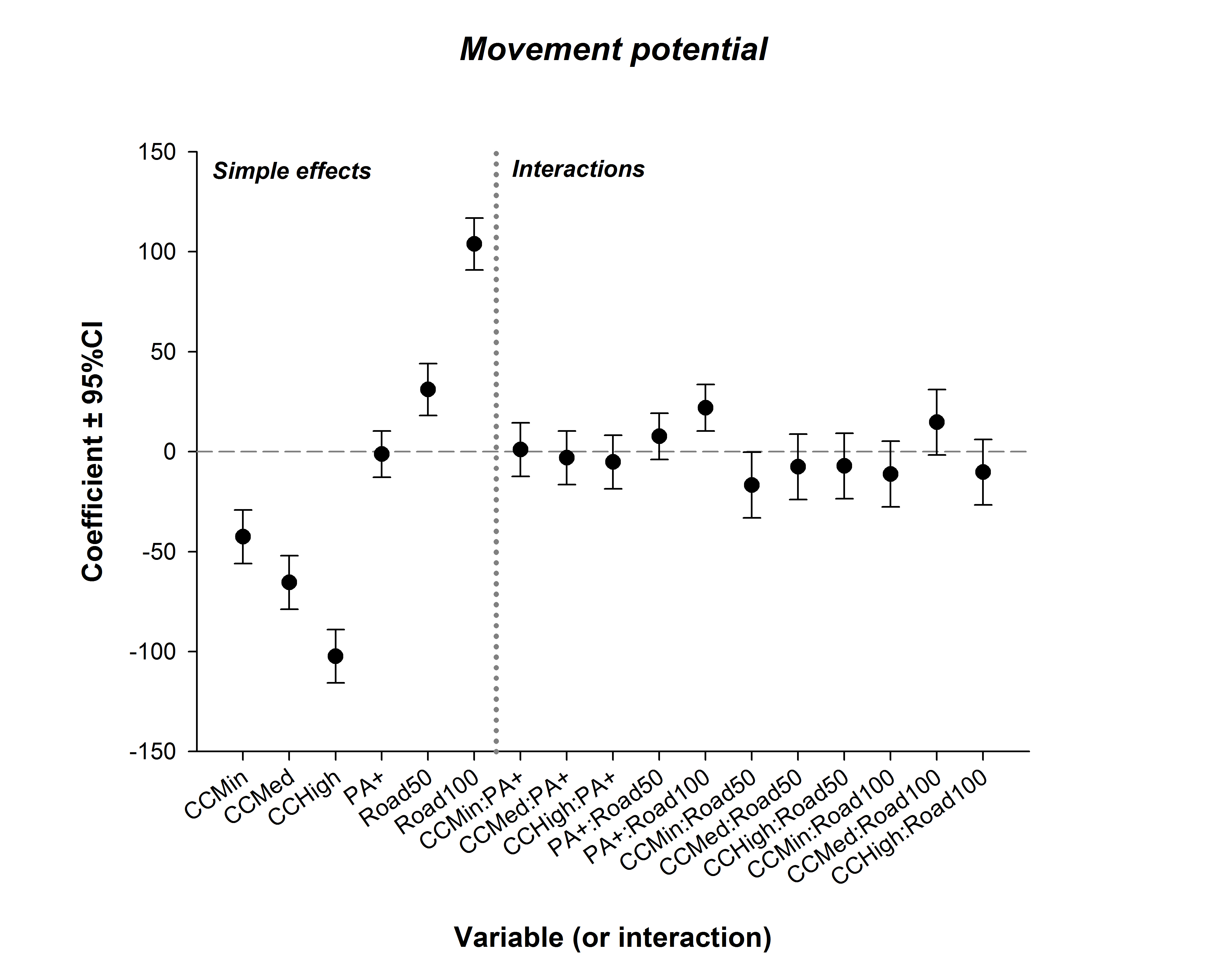

Supplement: Supplementary file 1 — Supplementary Information [file 41598_2018_34822_MOESM1_ESM.doc]
